# Absence of early platelet increment in healthy mice during decitabine treatment

**DOI:** 10.1038/s41598-022-26821-8

**Published:** 2022-12-23

**Authors:** Juliane Baumann, Markus Spindler, Yannick Throm, Michael Lübbert, Markus Bender

**Affiliations:** 1 Institute of Experimental Biomedicine – Chair I, University Hospital Würzburg, Josef-Schneider-Str. 2, 97080 Würzburg, Germany; 2 Department of Hematology, Oncology and Stem Cell Transplantation, Faculty of Medicine, University Medical Center Freiburg, University of Freiburg, Freiburg, Germany; 3 German Consortium for Translational Cancer Research, Freiburg, Germany

**Keywords:** Thrombopoiesis, Platelets

## Abstract

Treatment of myelodysplastic syndromes includes the administration of the hypomethylating agent decitabine. An early platelet response in decitabine-treated myelodysplastic syndrome patients is a predictor of overall survival. The effect of decitabine on megakaryocytes and the bone marrow, however, is understudied. We show that an early platelet increment was not detectable in healthy mice during decitabine treatment. Analyses of bone marrow sections revealed vessels with dilated lumina, decreased cellularity, but increased number of red blood cells and the presence of (pro)platelet-like particles. Taken together, decitabine treatment of healthy mice does not induce an early platelet increment, but affects the bone marrow.

## Introduction

Myelodysplastic syndromes (MDS) are clonal hematopoietic disorders with heterogeneous presentation ranging from ineffective hematopoiesis leading to peripheral blood cytopenias to variable rate of progression to acute myeloid leukemia^1,2^. Hypermethylation-induced gene silencing of tumor suppressor and other cancer-related genes plays an enormous role in tumor formation^3^. Demethylating agents are used in cancer treatment to reactivate those epigenetically silenced genes^4^. One therapeutic intervention for patients with MDS besides supportive care, hematopoietic growth factors, immunosuppressive therapies, and allogeneic stem cell transplantation, is the administration of decitabine (DAC; 5-aza-2′-deoxycytidine), which is a cytosine analogue that is incorporated into the DNA and inhibits DNA methyltransferases^3,5,6^. Hence, MDS patients with impaired maturation and differentiation of hematopoietic cells are treated with DAC to improve cytopenia^6,7^. However, hypomethylating agents have cytotoxic effects through inhibition of DNA synthesis and cell death at higher doses^8^. A predictive factor of a successful therapy and overall survival of MDS patients is an early platelet response (EPR) meaning an increase in platelet counts after DAC treatment^9,10^. Mice treated with clinically relevant doses of 15 mg/m^2^ DAC also displayed increased platelet counts already after 12 h^8,11^. Data from in vitro studies suggested enhanced megakaryocyte (MK) maturation and platelet release to be responsible for the increased platelet count in mice and human patients^11,12^. Recently, the analysis of bone marrow biopsies of MDS patients with EPR revealed more juvenile MKs before DAC treatment and more naked MKs, indicative for platelet shedding, after DAC treatment^13^. To better understand the impact of DAC on bone marrow MKs and the bone marrow compartment in vivo, we treated mice with DAC and examined bone marrow sections.

## Results

**Figure 1:**
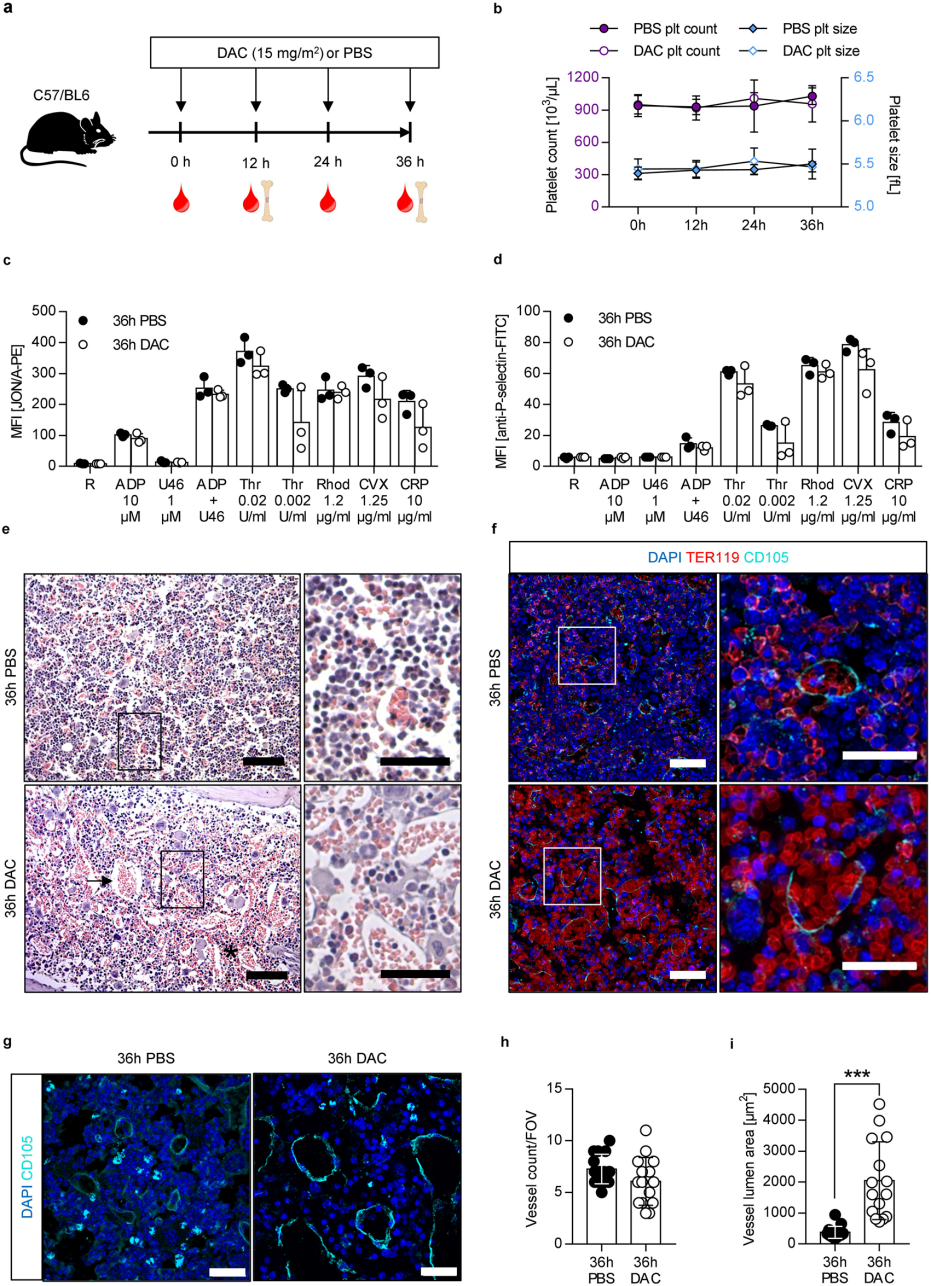
No EPR, but bleeding and vasodilation in the bone marrow of C57BL/6 mice after i.v. injection of DAC. (**a**) Experimental setup. C57BL/6 mice were injected every 12 h with 15 mg/m^2^ DAC. Blood and femura were collected at the indicated time points and subsequently analyzed. (**b**) Platelet count and size of PBS- or DAC-treated (15 mg/m^2^) mice were determined over time (0 h, 12 h, 24 h and 36 h) by a hematology analyzer. Mean ± s.d. of at least three mice is shown; the experiment was performed twice. (**c,d**) In vitro activation of platelets (*c* αIIbβ3 integrin activation, *d* P-selectin exposure) from DAC-treated and control mice; *R* resting, *ADP* adenosin diphosphate, *U46619 (U46)* thromboxane analogue, *Thr* thrombin, *Rhod* rhodocytin, *CVX* convulxin, *CRP* collagen related peptide. Each data point represents an individual mouse. (**e**) Representative hematoxylin/eosin staining of mouse femur after 36 h of treatment with PBS or DAC (15 mg/m^2^); scale bar represents 100 µm (left) and 50 µm (right). Asterisk indicates bleeding into bone marrow tissue; arrow indicates vasodilation. (**f**) Representative confocal images from bone marrow cryosections of PBS- and DAC-treated mice 36 h after treatment. Samples were stained for a vessel marker CD105 (cyan; CD105 is also expressed by macrophages and stem cells), red blood cell lineage marker TER119 (red) and with DAPI to visualize the nuclei (blue). Scale bar represents 50 µm in the overview images and 30 µm in the zoom-in images. (**g**) Representative confocal images from bone marrow cryosections of PBS- and DAC-treated mice 36 h after treatment. Specimens were stained for a vessel marker CD105 (cyan) and with DAPI to visualize the nuclei (blue). Scale bar represents 30 µm. (**h**) Quantification of vessel count per field of view (FOV). Each vessel segment at the edge of the image was treated as an individual vessel. (**i**) Vessel lumen area of the counted vessels in (**h**). (**h,i**) Each data point represents the vessel count (**h**) or mean vessel lumen area (**i**) of one image. In total 15 images (5 images per mouse) from 3 mice per condition were analyzed. (**b,c,d,h,i**) Values are mean ± s.d. of three mice per group. ***p ≤ 0.001.

**Figure 2:**
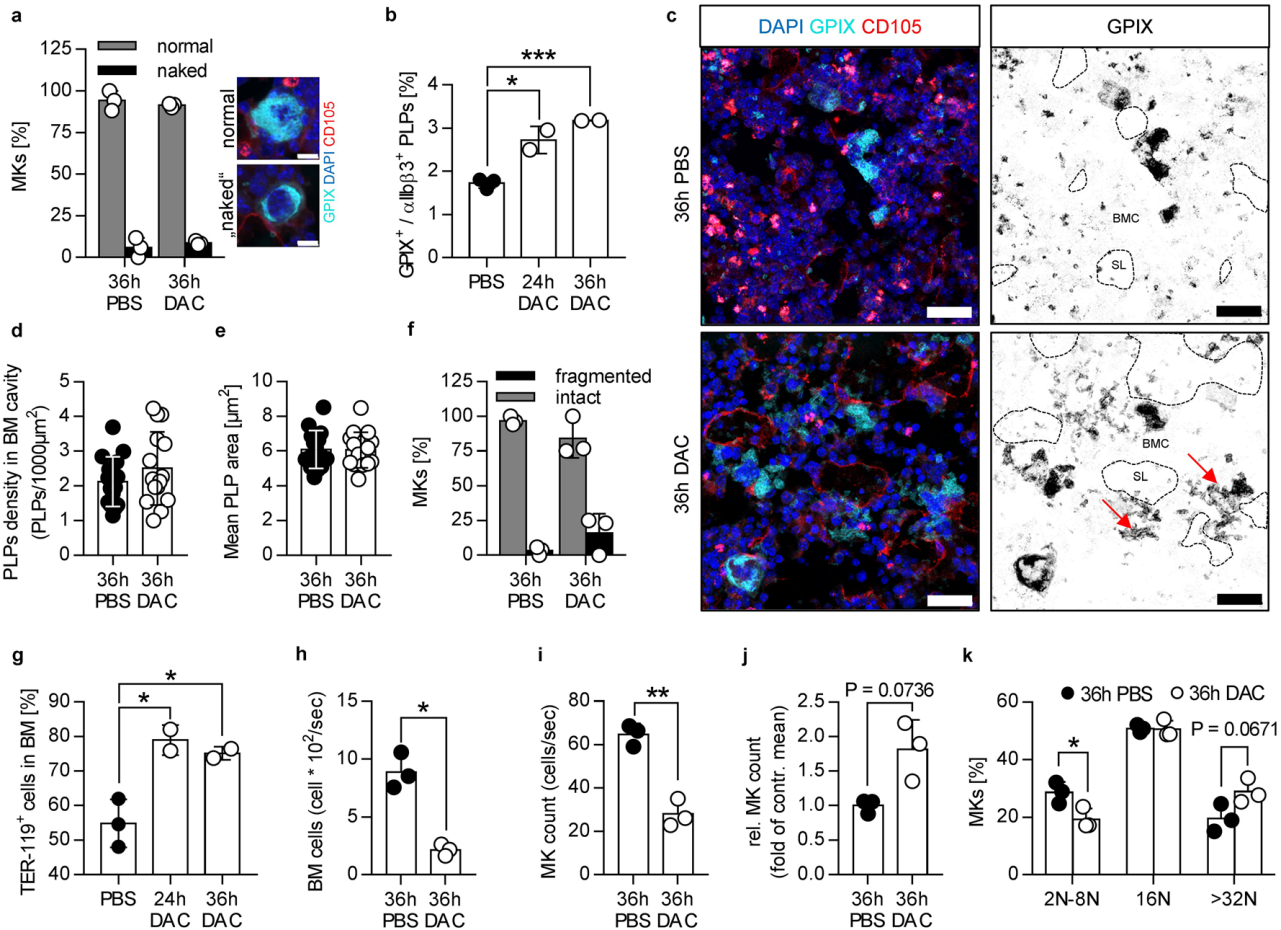
Reduced cellularity, elevated number of TER119 positive cells and overall unaltered MK ploidy in C57BL/6 mice after i.v. injection of DAC. (**a**) Quantification of the relative MK count in PBS- and DAC-treated mice. MKs were classified in “normal” (well developed nucleus and cytoplasm) and “naked” (well developed nucleus but less cytoplasm) MKs based on cryosections stained for GPIX (MKs), CD105 (vessel) and DAPI (nucleus). Representative images of a “normal” and a “naked” MK are shown on the right. Scale bar represents 10 µm. Each data point represents the mean value of MKs from one mouse (5 images per mouse). (**b**) Relative amount of platelet-like particles (PLPs, double positive events: GPIX^+^ and αIIbβ3^+^) in bone marrow samples from PBS- and DAC-treated mice after 24 h and 36 h. Each data point represents one mouse. Values are mean ± s.d. of at least two mice per group. *0.01 ≤ p < 0.05; ***p ≤ 0.001. (**c**) Representative confocal images from bone marrow cryosections of PBS- and DAC-treated mice 36 h after treatment. Specimens were probed for GPIX (MKs, cyan), CD105 (vessel, red) and DAPI (nucleus, blue). On the right only the GPIX channel is depicted as inverted image to visualize the PLPs in the bone marrow cavity (BMC). Vessels and sinusoidal lumen (SL) are indicated as dashed line based on the CD105 staining. The red arrows point to fragmented MKs in the bone marrow cavity. Scale bar represents 30 µm. (**d**) PLPs density in the bone marrow cavity based on the GPIX and CD105 staining. PLPs and vessels were segmented and the density per area bone marrow cavity calculated. Each data point represents the mean PLPs density in one image. In total 15 images (5 images per mouse) from 3 mice per condition were analyzed. (**e**) Mean PLP area based on GPIX staining. PLPs were segmented and the mean PLP area per images was calculated. In total 15 images (5 images per mouse) from 3 mice per condition were analyzed. (**f**) Quantification of the relative MK count in PBS- and DAC-treated mice. MKs were classified in intact and fragmented MKs based on the GPIX staining in (**c**). (**g**) Relative amount of TER119 positive cells in the bone marrow as measured by flow cytometry. Each data point represents one mouse. (**h–j**) Bone marrow cellularity measured by flow cytometry with a constant flow rate. Bone marrow cells were defined as SYTOX blue positive (nucleated) events after fixation and permeabilization. MKs in (**i**) were defined as GPIX positive events. The relative MK count was calculated as fold of control mean. (**k**) MK ploidy level was measured by GPIX and SYTOX blue staining via flow cytometry. (**f–k**) Each data point represents one mouse. Values are mean ± s.d. of at least three mice per group. *0.01 ≤ p < 0.05; **0.001 ≤ p < 0.01.

To address the impact of DAC on bone marrow MKs and the bone marrow compartment in vivo, C57BL/6 mice were injected every 12 h with the clinically relevant dose of 15 mg/m^2^ DAC (Fig. 1a), which has also been used in mice^7,11^. Unexpectedly, we did not observe an EPR in mice as previously reported^11^. Platelet count and size as well as platelet activation (integrin αIIbβ3 activation and P-selectin exposure) of C57BL/6 mice were not significantly altered over a time period of 36 h after DAC treatment (Fig. 1b–d). The platelet counts of DAC-treated mice were also comparable to controls at the early time point of 3 h (data not shown). Other blood parameters such as red and white blood cell count, hematocrit, and hemoglobin were also unaltered during the phase of DAC treatment (Supplementary Fig. 1). However, we detected increased bleeding into the bone marrow cavity indicative for impaired endothelial barrier function already after 12 h (Supplementary Fig. 2), which was more pronounced after 36 h (Fig. 1e). This was confirmed by immunostaining of TER119 on bone marrow cryosections showing elevated numbers of TER119 positive cells (erythrocytes and red blood cell progenitors) (Fig. 1f). DAC treatment also led to strongly dilated bone marrow sinusoids as observed at the 36 h time point (Fig. 1e–i). To test, whether a low concentration of DAC might induce EPR in mice, we injected only 3.5 mg/m^2^ into C57BL/6 mice (Supplementary Fig. 3a). However, a low concentration of DAC did also not change platelet count and platelet size in mice (Supplementary Fig. 3b), whereas we still observed increased bleeding into the bone marrow compartment (Supplementary Fig. 3c). As published earlier, biopsies of MDS patients with EPR after DAC treatment displayed an increased number of “naked” MKs with a large nucleus and less cytoplasm indicating high rates of platelet shedding^13^. Therefore, we stained bone marrow cryosections for GPIX (MK and platelet specific marker) and found no alteration in the percentage of “naked” MKs in DAC-treated mice compared to control mice (Fig. 2a), which is in line with the absence of EPR in mice (Fig. 1b). However, we detected a higher percentage of GPIX- and αIIbβ3-positive (pro)platelet-like particles (PLPs) at 24 and 36 h in samples of DAC-treated mice (Fig. 2b). The presence of PLPs in the bone marrow cavity was confirmed by immunostainings of cryosections probed for GPIX (Fig. 2c). In contrast to the flow cytometry data, image analyses revealed no significantly increased accumulation of PLPs in the BM cavity (Fig. 2d) and in addition a comparable PLP area in bone marrow of control and DAC-treated mice (Fig. 2e). The PLPs in the bone marrow cavity showed no obvious morphological signs of platelet activation. Therefore, these fragments resemble resting platelets supporting the possibility that they were ectopically released by MKs and not flushed from the circulation into the bone marrow cavity. In addition, we detected in bone marrow sections of DAC-treated mice moderately, but not significantly, elevated numbers of fragmented MKs (Fig. 2f). One possibility might be that these fragmented MKs could further fall apart during sample preparation resulting in elevated PLPs in the flow cytometry experiment (Fig. 2b). Furthermore, the PLP levels in the flow experiment were correlated to the bone marrow cell count and in the image based analysis to the bone marrow cavity area which might explain the different results. In addition, a larger population of TER-119 + cells after 24 and 36 h of DAC treatment was detectable by flow cytometry (Fig. 2g) confirming the data from histological- and cryosections (Fig. 1e,f). Overall, we observed a reduced cellularity in the bone marrow by flow cytometry (Fig. 2h) as well as by image analysis (Supplementary Fig. 4a–c), including a reduced total number of MKs in DAC-treated mice (Fig. 2i). However, there was a trend of increased relative MK counts compared to the total cellularity (P = 0.0736, Fig. 2j), suggesting a weaker effect of DAC on the susceptibility of the MK population. In line with this, we detected an overall comparable distribution of ploidy levels with only a slight decrease in percentage of immature MKs (2 N–8 N) and a trend to elevated numbers of mature MKs (> 32 N) in DAC-treated mice (Fig. 2k).

**Figure 3:**
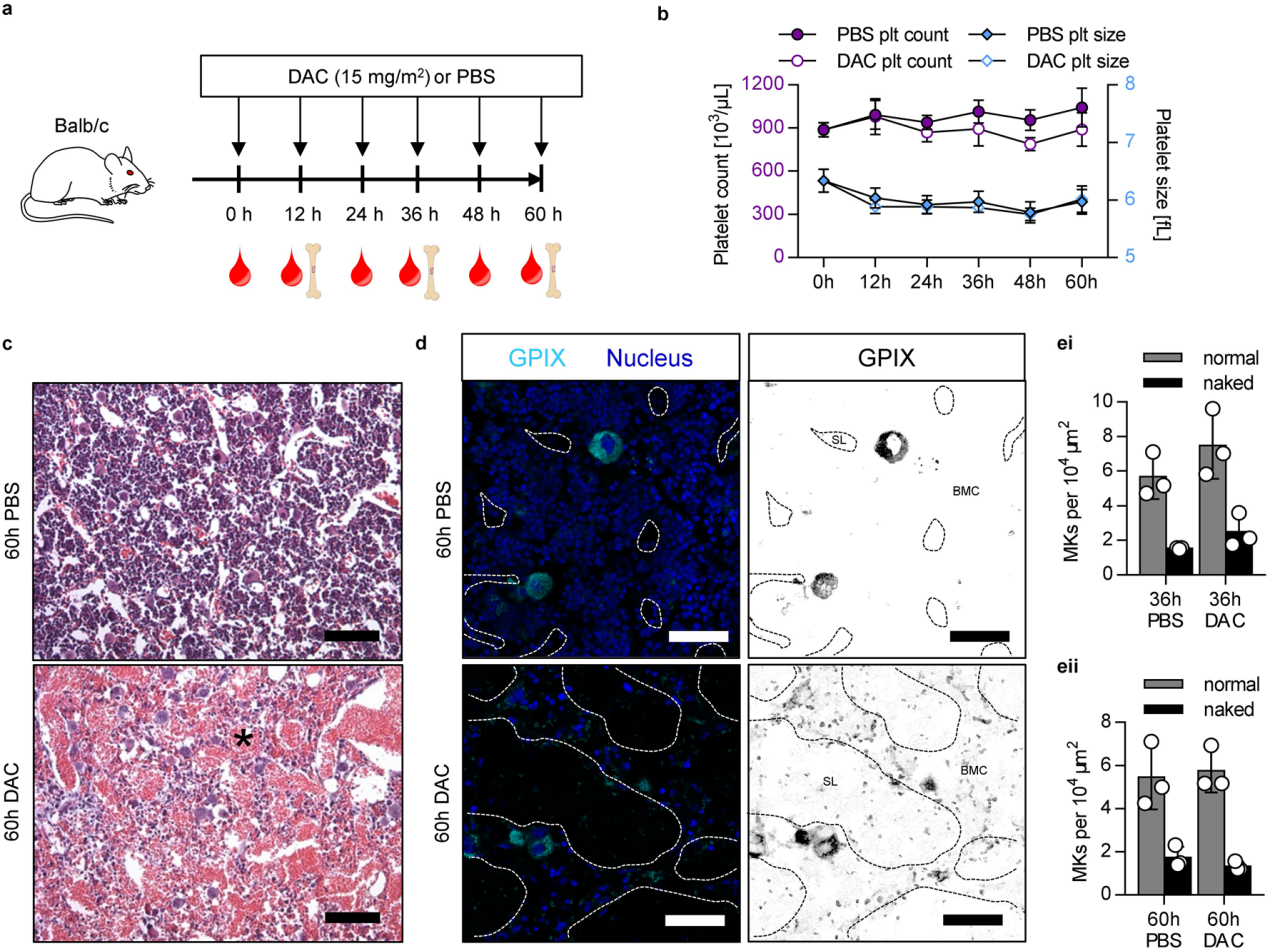
No EPR, but bleeding and vasodilation with PLPs in the bone marrow of Balb/c mice after i.v. injection of DAC. (**a**) Experimental setup. Balb/c mice were injected every 12 h with 15 mg/m^2^ DAC or PBS. (**b**) Platelet count and size of PBS- or DAC-treated (15 mg/m^2^) Balb/c mice were determined over time (0 h, 12 h, 24 h, 36 h, 48 h and 60 h) by a hematology analyzer. Values are mean ± s.d. of six mice. (**c**) Representative hematoxylin/eosin staining of mouse femur after 60 h of treatment with PBS or 15 mg/m^2^ DAC. Scale bar represents 100 µm. (**d**) Representative confocal images of bone marrow cryosections of PBS- and DAC-treated mice. Samples were stained for the specific MK/platelet marker GPIX (left: cyan; right: black) and with DAPI to visualize the nuclei (blue). Dashed white (left) and black (right) lines indicate vessels in the bone marrow based on CD105 staining (data not shown). Scale bar represents 50 µm. *BMC* bone marrow compartment, *SL* sinusodial lumen. (**c,d**) Images are representative for three mice per group. (**ei**, **eii**) Quantification of normal and naked MKs per 10,000 µm^2^ based on GPIX staining after 36 h (**ei**) and 60 h (**eii**) after DAC treatment. Three mice per condition and time point were analyzed.

Wang et al*.* observed an increase in platelet count in Balb/c mice already 12 h after DAC treatment^11^. To exclude the possibility that the different mouse background accounts for the different observations, we analyzed the platelet count and size as well as the bone marrow structure after treatment of Balb/c mice with 15 mg/m^2^ DAC (Fig. 3a). Nevertheless, we were also unable to detect an EPR over a time period of 60 h in DAC-treated Balb/c mice (Fig. 3b). Bleeding into the bone marrow cavity was also observed in Balb/c mice (Supplementary Fig. 5; 36 h; Fig. 3c: 60 h). Immunostaining on bone marrow cross-sections of intact femura revealed also the presence of PLPs (Fig. 3d) in Balb/c mice after DAC treatment. In line with our data from C57BL/6, we also found in Balb/c mice comparable numbers of “naked” and morphological normal MKs (Fig. 3ei: 36 h; eii: 60 h). Together, our data show that DAC treatment of mice does not result in an EPR, but leads to reduced cellularity, vasodilation and increased bleeding into the bone marrow cavity indicative for an impaired endothelial barrier function.

## Discussion

In this study, we demonstrate that i.v. injection of DAC into mice resulted in no EPR, but massively dilated bone marrow vessels and bleeding into the bone marrow cavity.

Hypomethylating agents such as DAC have been approved for MDS indications^6^. Some but not all MDS patients show an EPR after DAC treatment, which may be a useful early indicator for a favorable outcome^14^. The mechanism of how DAC increases the platelet count has remained elusive. The analysis of MK morphology in bone marrow biopsies of MDS patients with EPR after DAC treatment showed that a larger pool of MKs potentially underwent platelet shedding as more “naked” MKs were observed^13^. Another study showed a 30% platelet count increase in Balb/c mice 12 h after 15 mg/m^2^ i.v. injection of DAC, which was suggested to be the result of enhanced platelet release and MK maturation^11^. Unexpectedly, we could not measure an increase in platelet count after injection of 15 mg/m^2^ DAC or even at a lower dose (3.5 mg/m^2^) over an observation period of 60 h. We performed the experiments in C57BL/6 and Balb/c mice to exclude an effect of the mouse background. While we used the automated scil Vet abc Plus + Hematology analyzer to determine the platelet count, a haemacytometer was used in the other study^11^. However, the difference between our data and data from Wang and colleagues^11^ regarding the EPR in mice after DAC treatment remains unclear. DAC treatment is among others indicated in MDS patients with severe cytopenia, including thrombocytopenia^6^. The limitation of our study is that we used healthy mice with a basal platelet count of approximately 900,000 platelets per µL. This might be a possible explanation why we did not observe an EPR after DAC injection into mice. Additional studies will be needed using mouse models, which are engineered to recapitulate MDS characteristics.

Interestingly, patients with EPR showed an overall stable or even increased bone marrow cellularity after DAC treatment, whereas non-responsers showed reduced cellularity^13^. In agreement with the patient data, we observed reduced bone marrow cellularity in mice after DAC treatment. Whether the presence of PLPs and bleeding in the bone marrow compartment of mice is a consequence of the reduced bone marrow cellularity, the dilated vessels or in case of the PLPs an ectopic release from MKs remains to be explored. In contrast to our observation in mice, bleeding into the bone marrow cavity has not been observed in those patients.

Our data show that treatment of healthy mice with DAC does not induce EPR, as observed for MDS patients, but has an effect on the bone marrow in the absence of a clear myelotoxicity during the DAC treatment. Future studies are required to better understand this observation in mice.

## Methods

### Animals

Balb/c and C57BL/6 mice were obtained from Charles River. 7–8 weeks old female mice were used for this study. All animal studies were approved by the district government of Lower Franconia, Germany (license number 2-1087) and all methods were performed in accordance with the relevant guidelines and regulations. We followed the guidelines of ARRIVE (Animal Research: Reporting of In Vivo Experiments). Monitoring of mice revealed that their health and general conditions were not affected by decitabine treatment during the observation period of up to 60 h.

### DAC administration and blood collection

DAC was purchased from Sigma. DAC (3.5 or 15 mg/m^2^) or PBS was i.v. injected into mice and blood was collected retro-orbitally every 12 h in EDTA-tubes and analyzed using a scil Vet abc Plus + Hematology analyzer.

### Flow cytometry

Bone marrow from femora was isolated and incubated with the Fc receptor-blocking antibody (clone 2.4G2) and stained with anti-GPIX (FITC; emfret), anti-αIIbβ3 (PE; emfret) or anti-TER119 (APC; BioLegends) antibodies. Flow cytometric measurements were performed on a BD FACS Celesta. For ploidy measurement bone marrow cells were fixed and permeabilized in order to stain the nucleus with SYTOX blue. Anti-αIIbβ3 antibodies (Alexa Fluor 647 labeled; emfret) were used to identify MKs and progenitors. To analyze the cellularity of the bone marrow, samples were measured with a constant flow rate (60 µL/min) for 10 s and the cell count (SYTOX blue positive events) was then calculated to cells per sec.

### Hematoxylin and eosin staining of femora sections

Femora were fixed in 4% paraformaldehyde for 30 min and decalcified in 10% EDTA under rotation at room temperature for 7 days. Samples were dehydrated using the LEICA ASP200S and immediately embedded in paraffin. Finally, sections were rehydrated and stained with hematoxylin and eosin. Images were taken with a Leica DMI400B microscope.

### Immunostaining of femora sections

Femora were fixed in 4% paraformaldehyde for 30 min and cryoprotected in a graded sucrose series. Samples were embedded in Cryo-Gel (Section lab) and frozen at − 20 °C. Cryosections were generated using the Kawamoto method^15^ and subsequently stained for GPIX (MKs and PLPs; anti-GPIX antibody Alexa Fluor 488/Alexa Fluor 546/Alexa Fluor 647 from emfret), CD105 (vessel; anti-CD105 antibody Alexa Fluor 546/Alexa Fluor 647, clone MJ7/18), TER119 (red blood cells; anti-TER119 from biolegend), donkey anti-mouse IgG Cy5 and nuclei (DAPI included in embedding reagent Fluoroshield from Sigma). Samples were visualized on a Leica TCS SP8 confocal microscope (objective 40x/1.3 oil; objective 63x/1.4 oil).

### Image analysis

Image analysis was performed with Fiji. The vessel area was measured manually based on the CD105 staining in confocal images and area fraction was calculated. Each vessel segment at the edges of the image were considered as a vessel. The quantification of the cellularity in the bone marrow cavity was based on DAPI staining in confocal images. For the analysis the Fiji plugin StarDist 2D was used (model: versatile fluorescent nuclei and optimized postprocessing thresholds for the selected model)^16^. Objects > 10 µm^2^ were considered as nuclei. The detection of PLPs in the bone marrow compartment was performed with the trainable WEKA segmentation plugin from Fiji and the beforehand identified vessels. Objects > 2 µm^2^ and < 50 µm^2^ with a circularity between 0.5 and 1.0 were considered as PLPs. The PLPs density in the bone marrow cavity was calculated based on the bone marrow cavity area in every image.

### Data analysis

The presented results are mean ± standard deviation from one experiment with at least three mice per group if not stated otherwise. Differences between control and DAC-treated mice were statistically analyzed using the Unpaired *t* test. *P*-values < 0.05 were considered as statistically significant: *P < 0.05; **P < 0.01; ***P < 0.001.

## Supplementary Information

Supplementary Figures.

## Data Availability

The data generated and analyzed in this study are available from the corresponding author on reasonable request.
