# Supplementary material for: Absence of early platelet increment in healthy mice during decitabine treatment
Source: Sci Rep. 2022 Dec 23;12:22266. doi: 10.1038/s41598-022-26821-8 (PMC9789030; doi:10.1038/s41598-022-26821-8)
Supplement: Supplementary file 1 — Supplementary Figures. [file 41598_2022_26821_MOESM1_ESM.pdf]

SUPPLEMENTARY INFORMATION

Absence of early platelet increment in healthy mice during decitabine treatment

Juliane Baumann[1#], Markus Spindler[1#], Yannick Throm[1], Michael Lübbert[2,3], Markus Bender[1,*]

[1]Institute of Experimental Biomedicine – Chair I, University Hospital Würzburg, Würzburg, Germany

[2]Department of Hematology, Oncology and Stem Cell Transplantation, Faculty of Medicine, University Medical Center Freiburg, University of Freiburg, Freiburg, Germany

[3]German Consortium for Translational Cancer Research, Freiburg, Germany

# equal contribution

*Correspondence to:

Markus Bender
(Josef-Schneider-Str. 2, 97080 Würzburg; Bender_M1@ukw.de; phone: 0049 931-201 48328)

Supplemental Figures

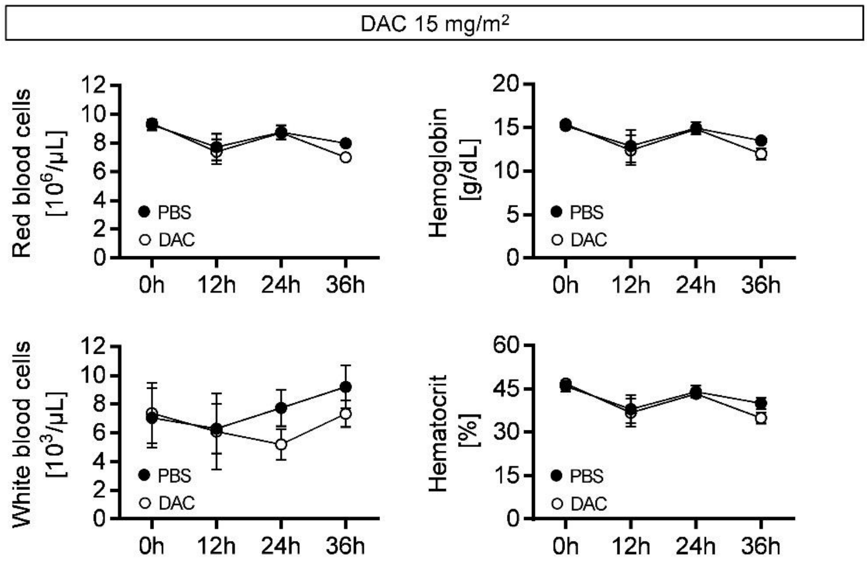

Supplemental Figure 1. Blood parameter of C57BL/6 mice after 0h, 12h, 24h and 36h of decitabine (15 mg/m^2; unfilled dots) and PBS (filled dots) treatment. Values are mean ± s.d. of at least 3 mice.

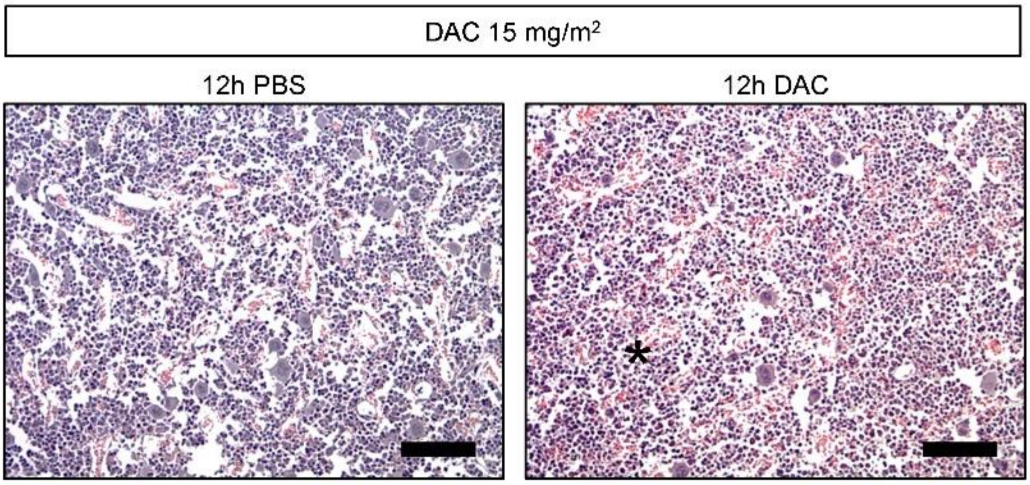

Supplemental Figure 2. Bleeding into the bone marrow of C57BL/6 mice after 12h of decitabine treatment (15 mg/m^2). Representative hematoxylin/eosin staining of mouse femur 12h after PBS or DAC treatment (15 mg/m^2). Scale bar represents 100 µm. Asterisk indicates bleeding into bone marrow tissue.

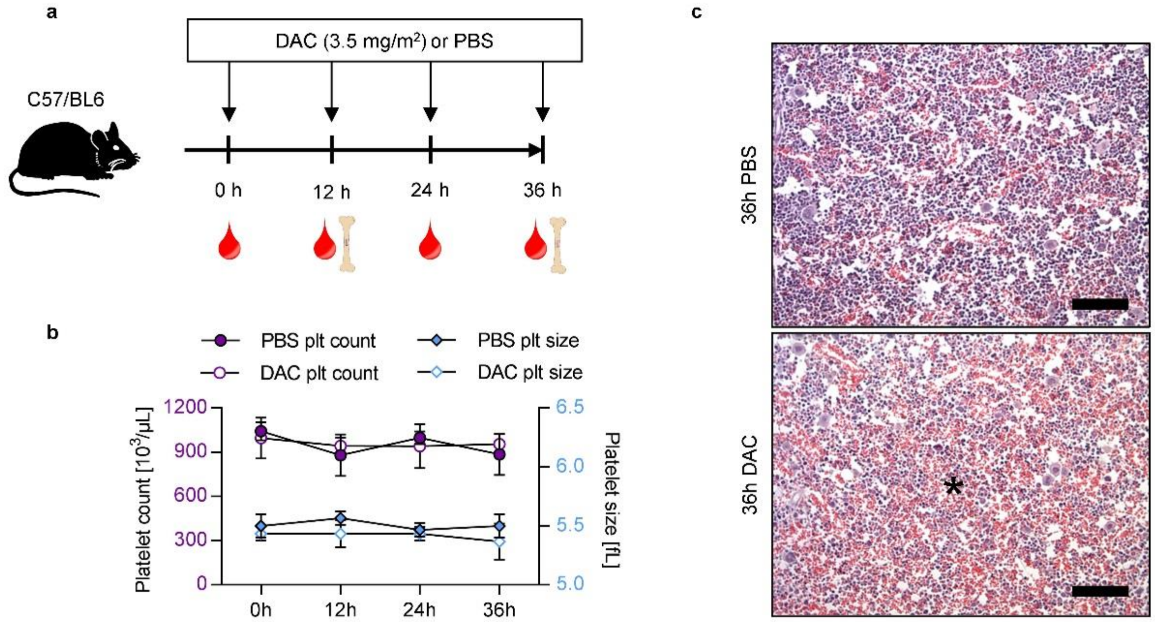

Supplemental Figure 3. No EPR, but bleeding into the bone marrow in low dose DAC-treated mice after i.v. injection. (a) Experimental setup. C57BL/6 mice were injected every 12h with 3.5 mg/m^2 DAC. Blood and femura were collected at the indicated time points and subsequently analyzed. (b) Platelet count and platelet size of PBS- or DAC-treated (3.5 mg/m^2) mice were determined over time (0h, 12h, 24h and 36h) by a hematology analyzer. Values are mean ± s.d. of at least three mice; the experiment was performed once. (c) Representative hematoxylin/eosin staining of mouse femur after 36h of treatment with PBS or DAC (3.5 mg/m^2); scale bar represents 100 µm. Asterisk indicates bleeding into the bone marrow.

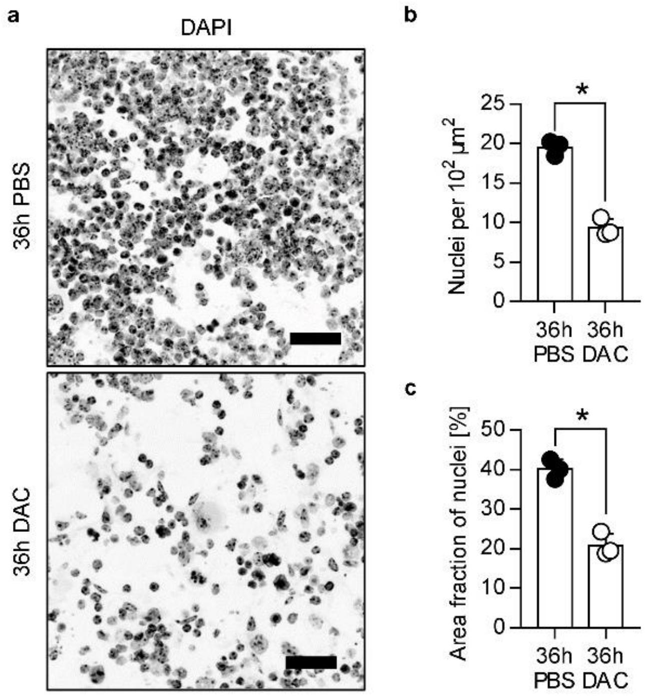

Supplemental Figure 4: Reduced cellularity in bone marrow of DAC-treated mice 36h after treatment. (a) Representative confocal images stained for nuclei (DAPI). The DAPI signal is depicted as inverted image for better visualization. Scale bar represents 30 µm. (b) Nuclei per area and (c) area fraction in % of nuclei on the whole image was calculated after nuclei segmentation with Fiji. Each data point represents the mean of 5 images per mouse. * $0.01 \leq p < 0.05$.

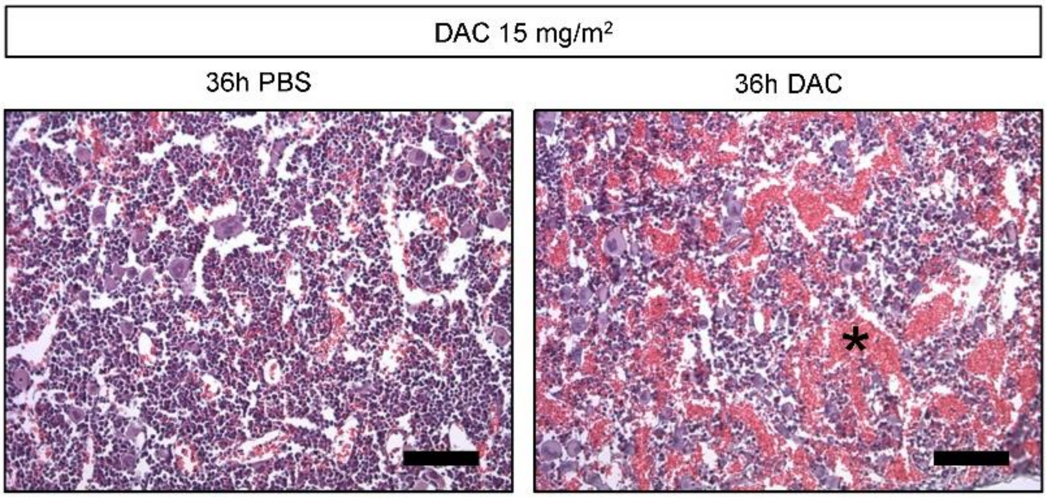

Supplemental Figure 5. Bleeding into the bone marrow of Balb/c mice after 36h of decitabine treatment (15 mg/m^2). Representative hematoxylin/eosin staining of mouse femur 36h after PBS or DAC treatment (15 mg/m^2); Scale bar represents 100 µm. Asterisk indicates bleeding into the bone marrow.
